# Development of an elution device for ViroCap virus filters

**DOI:** 10.1007/s10661-017-6258-y

**Published:** 2017-10-19

**Authors:** Christine Susan Fagnant, Matthew Toles, Nicolette Angela Zhou, Jacob Powell, John Adolphsen, Yifei Guan, Byron Ockerman, Jeffry Hiroshi Shirai, David S. Boyle, Igor Novosselov, John Scott Meschke

**Affiliations:** 1 Department of Environmental and Occupational Health Sciences, University of Washington, 4225 Roosevelt Way NE, Suite 100, Seattle, WA 98195 USA; 2 Department of Mechanical Engineering, University of Washington, Stevens Way, Box 352600, Seattle, WA 98195 USA; 3 PATH, 2201 Westlake Ave., Suite 200, Seattle, WA 98121 USA

**Keywords:** Virus elution, ViroCap filter, Electropositive filter, Environmental surveillance, Poliovirus, Wastewater

## Abstract

Environmental surveillance of waterborne pathogens is vital for monitoring the spread of diseases, and electropositive filters are frequently used for sampling wastewater and wastewater-impacted surface water. Viruses adsorbed to electropositive filters require elution prior to detection or quantification. Elution is typically facilitated by a peristaltic pump, although this requires a significant startup cost and does not include biosafety or cross-contamination considerations. These factors may pose a barrier for low-resource laboratories that aim to conduct environmental surveillance of viruses. The objective of this study was to develop a biologically enclosed, manually powered, low-cost device for effectively eluting from electropositive ViroCap™ virus filters. The elution device described here utilizes a non-electric bilge pump, instead of an electric peristaltic pump or a positive pressure vessel. The elution device also fully encloses liquids and aerosols that could contain biological organisms, thereby increasing biosafety. Moreover, all elution device components that are used in the biosafety cabinet are autoclavable, reducing cross-contamination potential. This device reduces costs of materials while maintaining convenience in terms of size and weight. With this new device, there is little sample volume loss due to device inefficiency, similar virus yields were demonstrated during seeded studies with poliovirus type 1, and the time to elute filters is similar to that required with the peristaltic pump. The efforts described here resulted in a novel, low-cost, manually powered elution device that can facilitate environmental surveillance of pathogens through effective virus recovery from ViroCap filters while maintaining the potential for adaptability to other cartridge filters.

**Electronic supplementary material:**

The online version of this article (10.1007/s10661-017-6258-y) contains supplementary material, which is available to authorized users.

## Introduction

Environmental surveillance (ES), or the process of sampling and analyzing environmental samples such as water, air, or surfaces, can provide key information regarding the presence and distribution of pathogens such as viruses. By sampling wastewater and wastewater-impacted surface water, viruses can be tracked with greater resolution. This enables a clearer understanding of vaccine coverage, viral distribution, and persistence, and therefore aids surveillance efforts when compared to clinical symptomatic observations alone (Battistone et al. 2014; La Rosa et al. 2014; Murray et al. 2013; World Health Organization 2015; Yanez et al. 2014; Cowger et al. 2017). For example, ES has shown the resurgence of wild poliovirus in previously documented polio-free areas (Anis et al. 2013; Manor et al. 2007; Manor et al. 1999; World Health Organization 2015, 2007) and has detected poliovirus presence prior to clinical detection (Cowger et al. 2017). Further, detection of viruses and bacteria in environmental waters can be used to indicate presence of human waste and subsequently deem a water source unsafe for recreational use and/or initiate remediation efforts (Betancourt et al. 2014). ES is also crucial for populations with silently circulating viruses (Fioretti et al. 2016; World Health Organization 2015), as early detection of viruses, such as poliovirus, can inform authorities working on vaccination, treatment, and infrastructure development efforts (Fumian et al. 2011; Hellmer et al. 2014; Lopalco 2017; Yanez et al. 2014; Asghar et al. 2014). Finally, in the instance of poliovirus, ES is key for declaring eradication, as it will help to ensure that the virus does not reemerge in the wild (Hovi et al. 2012; Lopalco 2017; World Health Organization 2015; Asghar et al. 2014; Cowger et al. 2017).

Positively charged filters, such as ViroCap™ and NanoCeram® filters, have many benefits for environmental surveillance of viruses. Unlike negatively charged filters, positively charged filters do not require conditioning of the water prior to filtration, making in-field filtration and filtration of large sample volumes feasible (Cashdollar and Wymer 2013; Fagnant et al. 2014; Fagnant et al. 2017b). Additionally, when ViroCap filters are used in conjunction with the bag-mediated filtration system, in-field gravity filtration of up to 10 L surface water and 3 L influent wastewater is feasible with no electrical power source (Fagnant et al. 2014; Fagnant et al. 2017b). ViroCap filters are commercially available, unlike glass wool filters, whose filter media is preconditioned and packed by hand (Ikner et al. 2012; Cashdollar and Wymer 2013). Commercial availability with specified tolerances simplifies quality assurance/quality control efforts, ensures a consistent product for reproducible results, and minimizes laboratory personnel time required for filter preparation. ViroCap and NanoCeram filter cartridges contain the same filter media (Cashdollar and Wymer 2013; Francy et al. 2013) but with different heights (e.g., 5.1 and 12.7 cm for ViroCap and NanoCeram, respectively), and the housings have different void spaces for eluate contact (100 and 500 mL, for ViroCap and NanoCeram, respectively) (Fagnant et al. 2014; Fagnant et al. 2017b). The 2″ (5.1 cm) ViroCap filter is shorter and encased in a smaller filter housing, which reduces the per-sample cost. The smaller size of the ViroCap filter also results in a lower elution volume and therefore a larger concentration factor (Fagnant et al. 2014). NanoCeram filters are part of the US Environmental Protection Agency (EPA) recommended method for virus monitoring from environmental and finished drinking waters (Fout et al. 2015), and their large surface area enables filtration of large water volumes (Karim et al. 2009). ViroCap and NanoCeram filters have been examined for their ability to concentrate viruses and bacteriophage from water, including adenovirus (Gibbons et al. 2010), bacteriophage MS2 (Bennett et al. 2010; Gibbons et al. 2010; Ikner et al. 2011), coxsackievirus B5 (Ikner et al. 2011; Karim et al. 2009), echovirus 1 (Ikner et al. 2011), echovirus 7 (Karim et al. 2009), norovirus (Karim et al. 2009), and poliovirus (Bennett et al. 2010; Fagnant et al. 2014; Ikner et al. 2011; Karim et al. 2009). Viral recovery rates with ViroCap and NanoCeram filters are generally similar to or better than the recovery rates obtained using other electropositive filters, such as glass wool and 1 MDS filters (Bennett et al. 2010; Fagnant et al. 2014; Karim et al. 2009; Soto-Beltran et al. 2013). Finally, ViroCap filters are also economical, easy to use, and field deployable (Cashdollar and Wymer 2013; Fagnant et al. 2014; Fagnant et al. 2017b). For this study, ViroCap filters were chosen for the reasons above, in addition to their higher poliovirus recovery rate when compared to NanoCeram filters (Fagnant et al. 2014).

Prior to detection or quantification, viruses adsorbed to electropositive filters must be eluted, often using a high protein, high pH solution (e.g., 1.5% beef extract, 0.05 M glycine, pH 9.5). Commonly, positive pressure is used to drive the elution process of electrostatic filters with the use of a pump (Berg et al. 1984; Francy et al. 2013; U.S. Environmental Protection Agency 2014) or a positive pressure vessel (Berg et al. 1984; Ikner et al. 2011; U.S. Environmental Protection Agency 2014). However, the use of peristaltic pumps and positive pressure can introduce biosafety and cross-contamination concerns, as peristaltic pumps cannot be autoclaved, and the setup between the pump, filter, and collection cup is not fully enclosed (Berg et al. 1984; U.S. Environmental Protection Agency 2014). Additionally, pumps and positive pressure vessels are expensive, their setup can be complex, involving multiple steps and/or components (Berg et al. 1984; U.S. Environmental Protection Agency 2014), and they require reliable mains electricity or a positive pressure source such as a compressed gas cylinder. Access to these systems can be challenging in resource-limited laboratories and in regions without a reliable power source. Further, depending on filter loading and pump strength, elution by a peristaltic pump can be slow and time-consuming. Finally, due to the lack of universal electrical socket design and voltage requirements, procurement of an appropriate peristaltic pump may be challenging based on differences in the distributor location and final destination.

The objective of this study was to develop a low-cost, manually powered ViroCap elution device for use in resource-poor laboratory environments. This is crucial for laboratories in low-income countries, as the high initial investment for single-purpose equipment can impede local laboratories from beginning ES efforts. A manually powered elution device for viral capture filters may potentially increase biosafety and remove the impediments created by elution with positive pressure sources, i.e., vessels and peristaltic pumps. This study describes a comparative analysis of the peristaltic pump method and two manually powered elution device designs for the effective elution of poliovirus from ViroCap filters.

## Methods

### Organism culture and enumeration

Stocks of the vaccine strain of poliovirus type 1 (PV1) were prepared by confluent lysis of buffalo green monkey kidney (BGMK) cell monolayers (Sobsey et al. 1978). Viruses were extracted with Vertrel XF (E. I. du Pont de Nemours and Company, Wilmington, DE, USA), and purified stocks were stored at − 80 °C (Mendez et al. 2000). Viruses were enumerated on 95% confluent BGMK cells with a previously described plaque assay modified to include an Avicel RC-581 (FMC Corporation, Philadelphia, PA, USA) overlay rather than agarose (Matrosovich et al. 2006; Sobsey et al. 1978). PV1 was provided by Mark Sobsey (University of North Carolina at Chapel Hill, Chapel Hill, NC, USA), and BGMK cells were provided by Daniel Dahling (USEPA, Cincinnati, OH, USA). Assays were performed in duplicate or triplicate on 9.5-cm^2^ wells using 200 μL aliquots of relevant dilutions. Infected cells were incubated at 37 °C and 5% CO_2_ for 48 h, then stained with 2% crystal violet in 20% methanol. Plaques were counted for enumeration of infectious virus. Viral recovery was calculated by dividing the recovered viral count by the seeded viral count.

Stocks of the bacteriophage MS2 (ATCC 15597-BI) were prepared by confluent lysis of *Escherichia coli* F-amp (ATCC 70081). Phages were extracted with Vertrel XF, and purified stocks were stored at − 80 °C. MS2 was enumerated by the previously described double agar layer on *E. coli* F-amp host (Adams 1959). Assays were performed in duplicate on 100-mm petri plates using 100 μL aliquots of relevant dilutions. Plates were incubated at 37 °C and for 18–20 h, and plaques were counted for infectious phage enumeration. MS2 recovery was calculated by dividing the recovered phage count by the seeded phage count.

Negative controls were also plated, including 1× phosphate-buffered saline (PBS), eluent (i.e., beef extract solution), and unseeded wastewater samples.

### ViroCap filter description

**Fig. 1:**
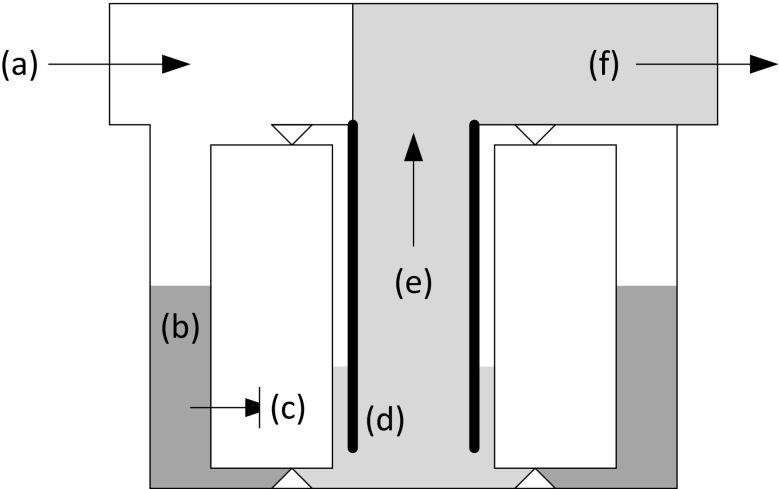
ViroCap filter. *Arrows* indicate fluid flow. Sample enters through inlet (*a*). Liquid outside filter cartridge is at relatively high level (*b*). Liquid passes through filter cartridge to relatively low level inside (*c*). Metal insert prevents air from entering liquid exiting filter cartridge (*d*). Liquid passes up filter cartridge by vacuum (*e*). Liquid exits through the top outlet (Fagnant et al. 2017b) (*f*)

Commercially available ViroCap filters (Scientific Methods, Granger, IN, USA) containing positively charged filter media were used in laboratory tests. The filter media has an average pore size of 2–3 μm and contains glass microfibers coated with alumina nanofibers (Bennett et al. 2010). The 70-mm-diameter ViroCap filter is 46 mm tall, with a total available surface area of 57,960 mm^2^. During filtration, water passes from outside the filter through the filter media and into the inner portion of the sealed filter cartridge (Fig. 1). Filter cartridges were secured in reusable filter housings, as previously described (Fagnant et al. 2017b). Briefly, the filter was seated in the housing sump body, and a lid with a 2–7/8″ long metal insert was screwed on to secure the filter in place. With this design, the liquid enters the filter housing through a side port inlet on the lid (Fig. 1 (a)). The liquid level outside the filter cartridge (Fig. 1 (b)) is always greater than the level inside the filter cartridge (Fig. 1 (c)) due to the metal insert (Fig. 1 (d)) that reaches from the lid (top of the filter housing assembly) to near the bottom of the filter housing. Thus, air cannot be blown into the inner liquid, and a low-pressure chamber is created. This causes the flow to be suctioned upwards (Fig. 1 (e)) and the liquid to then exit through a top outlet on the lid (Fig. 1 (f)). Prior to the incorporation of this metal insert, foaming and bioaerosol formation occurred (Fagnant et al. 2017b)*.*

### Sample preparation

Water was collected from West Point Wastewater Treatment Plant in Seattle, WA, USA. Ten liter grab samples of influent wastewater (after bar screens) were collected and stored at 4 °C until use, with a maximum storage time of 7 days. Samples were filtered through ViroCap filters at a rate of 2.0–0.2 L/min using a peristaltic pump (Masterflex L/S Precision Modular Drive; Cole-Parmer, Vernon Hills, IL, USA). Filters were stored at 4 °C for less than 24 h prior to elution. For viral recovery experiments, water samples were seeded with ~ 10^4^ PFU PV1 in 10 mL 1× PBS prior to filtration and eluted on the day of filtration.

### Elution

Filters were eluted inside of a biosafety cabinet by the addition of 100 mL 1.5% beef extract (BD Diagnostics, Sparks, MD, USA) and 0.05 M glycine (TCI America, Portland, OR, USA), at pH 9.5 to the filter inlet. A double elution was used for PV1 experiments, where the eluent was held in the filter for 15 min and then recovered through the filter outlet. An additional 100 mL eluent was added to the filter, left to stand 15 min, then recovered and combined with the first eluate. MS2 experiments were performed with a single elution, where the eluent was held in the filter for 30 min and then recovered through the filter outlet. Afterwards, the eluate was pH adjusted to 7.0–7.5 using 5 M HCl.

### Peristaltic pump

**Fig. 2:**
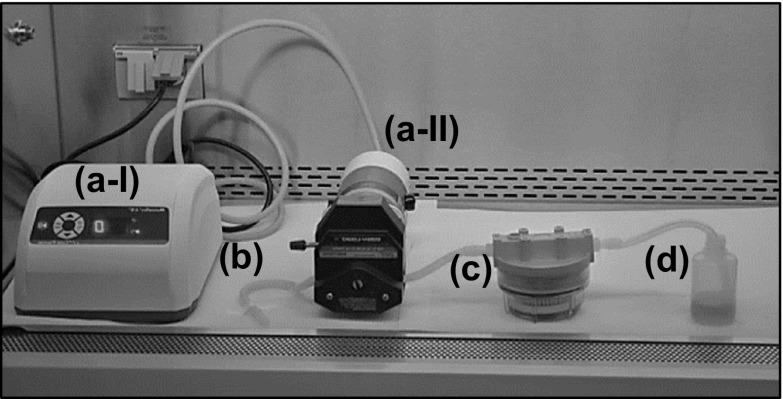
Peristaltic pump elution photo. Peristaltic pump controller (*a-I*), peristaltic pump (*a-II*), inlet tubing (*b*), ViroCap filter (*c*), and open collection bottle (*d*)

ViroCap elution was initially performed using a peristaltic pump (Fig. 2). Eluent was pulled from an open eluent cup through inlet tubing (Fig. 2 (b)) by a Masterflex L/S Precision Modular Drive (Cole-Parmer, Vernon Hills, IL, USA) peristaltic pump (Fig. 2 (a)) into the filter housing (Fig. 2 (c)) inlet at a rate of 0.4 L/min. After eluent contact time, positive pressure was applied, pumping air into the filter housing and driving the eluate out. Eluate was collected from the outlet in a 125-mL polypropylene collection cup (Fig. 2 (d)) that was open to the air. Autoclavable tubing was made of platinum-cured silicone. The peristaltic pump, which was not autoclavable, was located inside the biosafety cabinet.

### Elution device

**Fig. 3:**
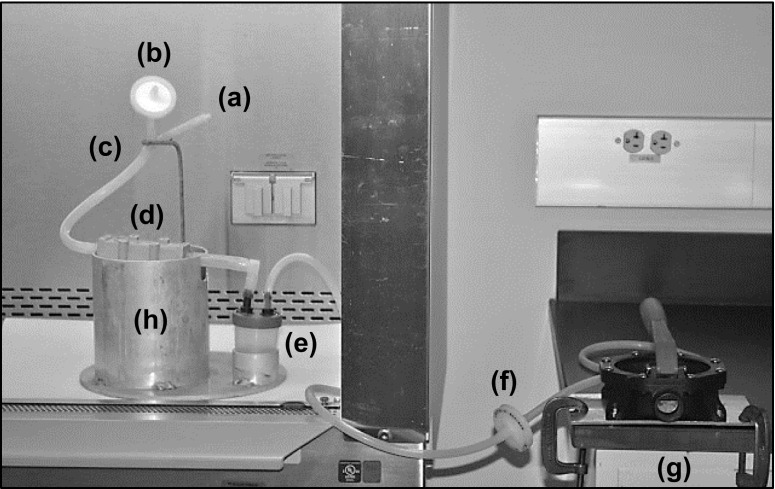
Elution device photo. Injection tubing inlet (*a*), hydrophobic filter (*b*), “Y”-tubing (*c*), ViroCap filter (*d*), collection cup (*e*), hydrophobic filter (*f*), bilge pump (*g*), and filter stand (*h*)

A manually powered elution device was developed to elute ViroCap filters (Fig. 3). The elution device was designed to improve biosafety and cross-contamination potential, while reducing cost and maintaining or improving usability. The eluent was injected into the ViroCap filter inlet using a syringe a Y-tubing system (Fig. 3 (c)), which was held in place with an eyelet stand. The base of the Y-tubing connected to the filter housing (Fig. 3 (d)) inlet and had a wall thickness of 0.125 in to prevent kinking of the tubing during eluent injection. The eluent was injected using a syringe via one branch (Fig. 3 (a)) of the Y-tubing. The other branch of the Y-tubing was fitted with a Vacu-Guard hydrophobic filter (Fig. 3 (b)) to prevent airlock. The Y reducer junction connected the three tubes and was made of nylon, which was chosen because it is autoclavable, corrosion resistant, water resistant, and inexpensive. To aid fluid flow, the filter was elevated and held stationary on an aluminum cylinder fitted with slots for the inlet and outlet ports to rest. During the eluent injection, the filter inlet was tilted upwards on the cylinder edge to aid fluid and air flow. After the eluent injection, the filter was returned to a horizontal position to enable the fluid to have full contact with the filter media. After eluent contact time, the eluate was drawn from the filter housing outlet into a 100-mL polypropylene collection cup fitted with a lid with two ports (Fig. 3 (e)) using a hand-operated bilge pump (Tenchchang 720GPH) (Fig. 3 (g)) to apply vacuum pressure. Similar to the Y-tubing, the tubing used to connect the filter housing outlet to the collection cup had a wall thickness of 0.125 in, which prevented kinking of the tubing during elution. Tubing connected the collection cup outlet port to a Vacu-Guard™ 50-mm disc hydrophobic filter (General Electric Healthcare, Chicago, USA) (Fig. 3 (f)) via a 30-cm length of tubing, and then to the bilge pump via a 300-cm length of tubing. A shorter length of tubing was used to connect the collection cup to the filter, which reduced the amount of material required sterilization and costs associated with tubing procurement and sterilization. All tubing was high-temperature silicone and autoclavable. An autoclavable aluminum plate stand (Fig. 3 (h)) consisted of the Y-tubing eyelet stand, the filter housing aluminum cylinder stand, and an aluminum collection cup holder, holding the filter housing and collection cup inside the biosafety cabinet. Aluminum was chosen for the filter stand due to its corrosion-resistant properties, ease of fabrication, low weight, and relatively low cost. In a typical laboratory setup, the bilge pump is attached to a bench outside the biosafety cabinet by two C-clamps and was not autoclavable.

### Secondary concentration and purification

After elution, PV1 samples were further concentrated using skimmed milk flocculation (Calgua et al. 2008). Briefly, 1 mL 5% skimmed milk (Oxoid Limited, Basingstoke, UK) was added to 100 mL samples, which were subsequently pH adjusted to 3.0–4.0. Samples were shaken for 2 h at room temperature and then centrifuged at 3500*×g* for 30 min. The pellet was resuspended in 10 mL 1× PBS, and then, 2.5 mL Vertrel XF was added. Samples were vortexed for 10 min and centrifuged at 3000*×g* for 15 min. The supernatant was retained, and the concentrated samples were then analyzed for PV1 as described above.

### Statistical analyses

Average absolute deviation from the mean was used to estimate error when the number of replicates (*n*) was too low to assume a normal distribution:

1 $$ AD=\frac{\varSigma \left|x-\overset{\acute{\mkern6mu}}{x}\right|}{n} $$

Unpaired Student’s or Welch’s *t* tests were used to compare recoveries under different conditions using Microsoft Excel 2016.

## Results and discussion

### Biosafety and cross-contamination potential

Two primary goals in the elution device development were to increase biosafety and reduce cross-contamination potential; these were addressed by making several modifications to the initial peristaltic pump elution technique. The first modification involved switching from a peristaltic pump to a bilge pump. The peristaltic pump (Fig. 2 (b)) applied positive pressure to the ViroCap filter inlet, which resulted in compressed air being released from the filter inlet into the air after elution. This could potentially introduce contamination and harmful bioaerosols into the biosafety cabinet (Walls et al. 2014). Therefore, replacement of the peristaltic pump with a bilge pump eliminated this risk as the bilge pump applied negative pressure from the filter outlet (Fig. 3 (g)). However, due to this change, a modification in the eluent injection method was required. To prevent vapor lock during the eluent injection into the filter inlet, a vent was necessary. The eluent injection system enclosed biological material inside the elution device system with a Y-tubing (Fig. 3 (c)). The Y-tubing had three prongs: (1) a top branch contained a luer lock adapter that the eluent injection syringe attached to (Fig. 3 (a)), (2) the second branch attached to a hydrophobic filter (Fig. 3 (b)) to aid air flow, and (3) the base prong connected to the filter inlet. The addition of the hydrophobic filter to the Y-tubing prevented vapor lock during the eluent injection while reducing cross-contamination potential.

Biosafety and cross-contamination concerns were also addressed by modifying the eluate collection receptacle (Table 1). Using a peristaltic pump, the eluate was collected in a bottle with an open top (Fig. 2 (d)). The eluate can foam, and popped bubbles can be released into the air, potentially bioaerosolizing viruses (Walls et al. 2014). For the elution device, the open collection bottle was replaced with a 100-mL collection cup fitted with a two-ported lid (Fig. 3 (e)). One port connected to the ViroCap filter outlet and the other to the bilge pump. Bioaerosols produced during elution were contained inside the lidded collection cup, and a hydrophobic filter (Fig. 3 (f)) prevented bioaerosols from entering the bilge pump.

**Table 1:** Comparison of ViroCap filter elution using a peristaltic pump and manual bilge pump (elution device) method

Variable	Peristaltic pump	Elution device
Biosafety and cross-contamination potential		
Receiving vessel	Open 125-mL bottle	100-mL specimen container
Non-autoclavable parts in BSC	Peristaltic pump	None
Use and specifications		
Setup time (min:s)	1:15	1:41
Active elution time (min)	23	26
Power mechanism	Electric	Manual
Weight (kg)	8.0	4.1
Surface area inside BSC (cm^2^)	840	510
Surface area outside BSC (cm^2^)	0	260
Height (cm)	16	29
Cost		
Materials and manufacturing cost for 10 samples^a^	~ US$1800	~ US$505–$555/$650–$750^b,c^
Efficiency and recovery		
PV1 yield, ± AD (%)	17.1 ± 4.6 (*n* = 6)	17.3 ± 2.3 (*n* = 6)
Max pump pressure drop (mmHg)	N/A	72 (1.4 psi)
Average elution volume, ± AD (mL)	100 ± 6.0 (*n* = 6)	97 ± 5.2 (*n* = 6)

*BSC* biosafety cabinet, *PV1* poliovirus type 1, *N/A* not applicable, *AD* average deviation, *n* number, *psi* pounds per square inch

^a^Assumes that reusable supplies (e.g., tubing, collection cups) are not disinfected between samples, and 10 complete sets of supplies are included in the cost

^b^Production at scale/single unit

^c^Range provides minimum and maximum estimated values

Finally, the elution device design was modified to allow all items in the biosafety cabinet to be sterilized (Table 1). Both the peristaltic pump and bilge pump have uneven surfaces, difficult-to-reach components, and internal parts that render full chemical surface disinfection challenging, and neither is autoclavable. Therefore, the bilge pump was physically separated from the elution device filter stand (Fig. 3 (h)) and placed outside the biosafety cabinet. This allowed all components inside the biosafety cabinet to be autoclaved, as they were heat tolerant (121 °C) and corrosion resistant. The elution device filter stand (26-cm diameter, 29 cm high) fits inside a standard biosafety cabinet and inside many standard top-loading autoclaves (Table 1). By placing the bilge pump outside the biosafety cabinet, its exposure to potential contaminants is greatly minimized.

### Cost

The cost of the elution device is reduced when compared to the peristaltic pump method (Table 1). The peristaltic pump method costs approximately US$1800, which includes the peristaltic pump, controller, electrical transformer, and 10 replacement sets of tubing, eluate cups, and collection cups. These components are reusable, although the tubing wears out over time due to the peristaltic pump, requiring periodic replacement. In contrast, the elution device was developed in a prototyping laboratory in Seattle, USA, and the cost estimates for the device range from $650–$750 as a single unit or $505–$555 at scale when produced in this type of environment. These costs may vary when produced in other settings and/or locations. This cost includes the supplies (bilge pump, filter stand, syringe, and 10 replacement sets of tubing, collection cups with ported lids, and Vacu-Guard filters) as well as the manufacturing costs. The supplies cost $450 as a single unit or $380 at scale (Online Resource 1), and the only non-autoclavable components of the elution device exposed in the biosafety cabinet are the hydrophobic filters and the eluent injection syringe. However, these items can be reused unless contamination occurs, and replacements cost approximately $18 and $10 each, respectively (Online Resource 1). The costs associated with the manufacturing of the elution device range from $200 to $300 for a single unit or $125–$175 at scale; steps associated with assembly are detailed in Online Resource 2. The filter stand and ported lid for the collection cup are custom fabricated, requiring waterjets, milling, and welding, with a total estimated time of 100–150 min for a single unit or 70–90 min when manufactured at scale.

In conjunction with an in-field, electricity-free filtration system such as the bag mediated filtration system (Fagnant et al. 2017b; Fagnant et al. 2014), a low-cost ViroCap filter elution system such as the elution device may enable laboratories to conduct virus elution work with reduced financial outlay. In addition, such a setup could facilitate virology research in laboratories that do not specialize in, or are just entering, the field of environmental surveillance and allow small scale or proof-of-concept data collection without large investments into single-purpose equipment. Also, the low cost of the elution device reduces the risk and cost of failure. A peristaltic pump is a piece of equipment that cannot be repaired easily if failure occurred. Failure could result in the need for complete replacement (approximately US$1500). In contrast, replacement of the bilge pump is more cost-effective as each costs approximately US$30.

### Operation and specifications

The usage of the elution device remained similar to or improved upon that of the peristaltic pump method. The overall time to set up the system required 75 s with the peristaltic pump and 101 s using the elution device (Table 1). Additionally, the active time required for the double elution method (as described above) using a peristaltic pump took an average of 23-min active personnel time per filter, while the elution device required an average of 26 min (Table 1). The overall time to set up the system required 75 s with the peristaltic pump and 101 s using the elution device (Table 1). This similar time requirement indicates a similar level of operational complexity between the two methods. Additionally, the elution device did not require electricity (Table 1), eliminating the need for adequate mains electricity and concerns associated with different international electrical outlets and voltages. This feature makes the elution device ideal for use in laboratories in low-resource settings with limited access to electricity.

The operation of the elution device was aided by the filter housing stand. The stand (Fig. 3 (h)) stabilizes the ViroCap filter housing with two “L”-shaped channels on opposite sides of the aluminum cylinder that holds the ViroCap filter housing. These channels allow the filter to securely rest in two positions: tilted for the eluent injection and flat for eluate contact. This improved stand is also very robust, as all components are made of metal and connected securely to the base plate.

Many of the physical design characteristics of the elution device were improved compared to those of the peristaltic pump. The weight of the elution device system is substantially less than that of the peristaltic pump system (Table 1). As the elution device system is relatively small and lightweight, this eases set up and take down, which may occur frequently in a laboratory without a biosafety cabinet dedicated to the elution of ViroCap filters. The surface area is comparable between the two systems, although the elution device footprint inside the biosafety cabinet is smaller than that of the peristaltic pump setup (Table 1). Furthermore, the filter stand of the elution device provides a better filter elution setup as compared to the system using the peristaltic pump, because all of the components for this device have designated locations for a specific placement on the filter stand (Fig. 3 (h)). The peristaltic pump method has resulted in occasional accidental sample spillage during pumping because the collection cup was not firmly secured. Finally, although the elution device is taller than the peristaltic pump method, it is easily placed in a standard biosafety cabinet after removal of the detachable eyelet stand that holds the Y-tubing in place.

### Efficiency and recovery

The elution device successfully moved eluate through filters that had processed 6 L raw wastewater, by creating a pressure drop of 72 mmHg. Filters eluted by the elution device had a comparable eluate volume recovery and virus yield to filters eluted by the peristaltic pump method, indicating that the ViroCap elution method did not affect eluate recovery efficiency (Table 1). The eluate volume recovery was not statistically significantly different between the peristaltic pump and elution device method (*p* = 0.66, *t* test), indicating that there was little sample volume loss due to device inefficiency.

Recovery of PV1 was measured from ViroCap filters after elution by the peristaltic pump or elution device, followed by secondary concentration using skimmed milk flocculation. Average PV1 recovery from the peristaltic pump and elution device measured 17.1% from the peristaltic pump and 17.3%, respectively, and the results between elution methods were not statistically significantly different (*p* = 0.92, *t* test) (Table 1). A similar effect was seen for MS2, where use of the peristaltic pump and elution device resulted in an average recovery of 145.5 and 126.6%, respectively, and no statistical difference was observed between results (*p* = 0.62, *t* test). MS2 recovery over 100% is likely due to disaggregation of the bacteriophage stock during processing (Fagnant et al. 2017a). These data indicate that use of the elution device instead of a peristaltic pump does not affect PV1 or MS2 recovery from ViroCap filters.

Additionally, the low levels of variation in virus recovery seen by Calgua et al. (2013) demonstrated the ability of the skimmed milk flocculation method to produce replicable results and indicated that the method is unlikely to significantly impacts results from this study. Results from this previous study show that skimmed milk flocculation yields approximately 50% virus recovery with a coefficient of variation of 12.2, 15.9, and 17.4% for JC polyomavirus, human adenovirus, and norovirus genogroup II, respectively (Calgua et al. 2013).

## Conclusions

Efforts described here resulted in a novel, low-cost, manually powered ViroCap elution device designed specifically for use in resource-limited laboratory environments. Its design incorporates features for improved biosafety and reduced likelihood of cross-contamination. These include a manually powered bilge pump that moves liquid through the ViroCap filter using negative pressure (in contrast to the positive pressure applied by the peristaltic pump), and all of the reusable items in the biosafety cabinet are autoclavable and fully enclose biological liquids and bioaerosols inside the elution system components. The manual device also maintains similar use of existing methods while maintaining convenience by reducing size and weight. When considering the much lower cost and improved biocontainment of the elution device, it presents a viable alternative to a peristaltic pump for effective, economical, and safe elution of ViroCap filters. While this study focused on elution of ViroCap filters, the economic and biosafety advantages of this device could be leveraged toward other uses. Future studies should explore adapting the developed device for the elution of other cartridge filters such as NanoCeram filters or Envirochek® cryptosporidium filters.

## Electronic supplementary material

ESM 1 (XLSX 13 kb)

ESM 2 (XLSX 12 kb)
